# BLACK CAREGIVERS’ CULTURAL, RELIGIOUS, AND SYMPTOM MANAGEMENT EXPERIENCES WITH HOME HOSPICE CARE

**DOI:** 10.1093/geroni/igad104.2466

**Published:** 2023-12-21

**Authors:** Taeyoung Park, Danetta Sloan, Dulce Maria Cruz-Oliver, M Cary Reid, Sara Czaja, Ronald Adelman, Ritchell Dignam, Veerawat Phongtankuel

**Affiliations:** Weill Cornell Medicine, New York City, New York, United States; Johns Hopkins Bloomberg School of Public Health, Baltimore, Maryland, United States; Johns Hopkins Hospital, Baltimore, Maryland, United States; Weill Cornell Medicine, New York City, New York, United States; Weill Cornell Medicine, New York City, New York, United States; Weill Cornell Medicine, New York City, New York, United States; VNS Health, New York City, New York, United States; Weill Medical College of Cornell University, New York City, New York, United States

## Abstract

Informal Black or African American (Black/AA) caregivers are at high risk for caregiver burden due to both greater caregiving responsibilities and unmet needs. However, there has been minimal research on the challenges Black/AA caregivers face after hospice enrollment. This study seeks to address this knowledge gap by applying qualitative methods to understand Black/AA caregivers’ experiences around cultural, religious, and symptom management challenges during home hospice care. Data from semi-structured interviews with 11 bereaved Black/AA caregivers of patients who received home hospice care were qualitatively analyzed. Cultural needs (e.g., knowing their language, having familiarity with foods) were perceived as not on top of mind for many Black/AA caregivers. However, there was concern of stigma around mental health preventing care recipients from sharing their mental health concerns and seeking resources. Many caregivers relied on their personal religious networks rather than services provided by hospice chaplains. Regarding symptom management, caregivers struggled most with managing patients’ pain, lack of appetite, and decline near End of Life (EoL). Lastly, caregivers reported increased burden during this phase of caregiving but were satisfied with the overall hospice experience. Our results suggest that tailored approaches that target mental health stigma in the Black/AA community and reduce caregiver distress around EoL symptoms may improve hospice outcomes among Black/AA hospice caregivers. Hospice spiritual services should consider offering services complementary to caregivers’ existing religious networks. Larger quantitative studies are needed to examine the clinical implications of these results in terms of patient, caregiver and hospice outcomes.

